# Surface Effects and Challenges for Application of Piezoelectric Langasite Substrates in Surface Acoustic Wave Devices Caused by High Temperature Annealing under High Vacuum

**DOI:** 10.3390/ma8125497

**Published:** 2015-12-19

**Authors:** Marietta Seifert, Gayatri K Rane, Benjamin Kirbus, Siegfried B Menzel, Thomas Gemming

**Affiliations:** IFW Dresden, SAWLab Saxony, PO Box 270116, 01171 Dresden, Germany; g.k.rane@ifw-dresden.de (G.K.R.); b.kirbus@ifw-dresden.de (B.K.); s.menzel@ifw-dresden.de (S.B.M.); t.gemming@ifw-dresden.de (T.G.)

**Keywords:** LGS, Langasite, RuAl, high-temperature, Surface Acoustic Waves, thin film

## Abstract

Substrate materials that are high-temperature stable are essential for sensor devices which are applied at high temperatures. Although langasite is suggested as such a material, severe O and Ga diffusion into an O-affine deposited film was observed during annealing at high temperatures under vacuum conditions, leading to a damage of the metallization as well as a change of the properties of the substrate and finally to a failure of the device. Therefore, annealing of bare LGS (La${}_{3}$Ga${}_{5}$SiO${}_{14}$) substrates at 800 ${}^{\circ }$C under high vacuum conditions is performed to analyze whether this pretreatment improves the suitability and stability of this material for high temperature applications in vacuum. To reveal the influence of the pretreatment on the subsequently deposited metallization, RuAl thin films are used as they are known to oxidize on LGS at high temperatures. A local study of the pretreated and metallized substrates using transmission electron microscopy reveals strong modification of the substrate surface. Micro cracks are visible. The composition of the substrate is strongly altered at those regions. Severe challenges for the application of LGS substrates under high-temperature vacuum conditions arise from these substrate damages, revealing that the pretreatment does not improve the applicability.

## 1. Introduction

During the last years, the piezoelectric substrate LGS (La${}_{3}$Ga${}_{5}$SiO${}_{14}$) was highly discussed for high temperature application in particular for surface acoustic wave (SAW) devices. The suitability of this substrate for SAW systems operating at higher temperatures was partially examined by several authors and promising results have been achieved [1,2,3,4]. Although LGS was reported to be temperature stable up to its melting point of 1470 ${}^{\circ }$C over the last few years, several groups revealed the diffusion of O and Ga out of the substrate during high temperature treatment [5,6,7]. If a film which contains elements with a high affinity to O is deposited, e.g., as an electrode on such a substrate severe oxidation of the film will take place also upon heating under vacuum as was observed for RuAl thin films at 800 ${}^{\circ }$C [7]. For these samples, which were deposited as a RuAl alloy film, a thick Al${}_{2}$O${}_{3}$ layer was formed on top of the substrate and a nearly pure Ru film remained above it. The presence of Ga within the RuAl film after annealing at 800 ${}^{\circ }$C has also been proven [7].

One attempt to avoid the diffusion of O and Ga into the electrodes is the addition of a diffusion barrier between substrate and film, e.g., Al${}_{2}$O${}_{3}$ [8]. Besides this idea, a preheating of the substrate is one approach to reach an equilibrium state with a reduced O diffusion. Moulzolf, *et al.* also shortly mention in their paper an experimental attempt with pre-annealed LGS substrates which led to a pronounced failure of their films after annealing, however, they do not comment on the annealing times and conditions of the LGS substrates; only the temperature of 850 ${}^{\circ }$C is mentioned [8].

Annealing effects of LGS under air conditions have already been reported [9]. The authors found that heating substrates in air for one week did not lead to obvious changes up to annealing temperatures of 1100 ${}^{\circ }$C, but that a temperature treatment at 1200 ${}^{\circ }$C for the same time span led to flaws at the substrate surface, where a huge change in composition (only 1/10 of the nominal content of Ga) was found. They suggest the formation of a lanthanum-silicon oxide compound [9].

Bardong, *et al.* investigated the influence of packaging atmospheres on the durability of high-temperature SAW sensors using LGS substrates among others [10]. They found no color change of the substrate during heating under air atmosphere, an effect which occurs during heating in vacuum, but in both cases there was a degradation of the crystal surface. Annealing the substrates under high vacuum conditions leads to a strong O gradient between sample and the surrounding and to a driving force for the O diffusion towards the free LGS surface, which is significantly reduced if the heating is performed under air. On heating under light vacuum conditions (0.4 mbar) at 650 ${}^{\circ }$C, oxygen and gallium oxide (Ga${}_{2}$O${}_{3}$) escape from the substrate [10].

The annealing experiments summarized above dealt either with the question whether LGS can really be applied up to its melting temperature [9] or with the influence of the packaging atmosphere on a SAW device at elevated temperatures [10]. A remaining open question is whether a pre-treatment has a positive influence on the high-temperature stability of the substrate and whether a metallization deposited on a pre-annealed substrate is less oxidized and therefore more stable. To answer these questions, bare LGS substrates have been annealed under high vacuum conditions ($p<10^{-5}$ mbar) at 800 ${}^{\circ }$C. As a metallization the RuAl alloy is used because of its interesting potential high temperature applications due to its high melting point and also since former experiments with untreated LGS substrates showed a complete oxidation of the Al within the film [7]. A comparison showing the influence of the pretreatment of the substrates is provided in this paper.

## 2. Experimental Section

Bare LGS substrates (FOMOS, 138.5${}^{\circ }$ Y-cut (Y-cut: the crystal is rotated around the *x*-axis by 138.5${}^{\circ }$ counterclockwise so that there is a new *y*′ direction. The crystal is now cut in that way, that the new *y*′ axis is the normal to the wafer surface.)) were annealed at 800 ${}^{\circ }$C separately for 10 h and 48 h under high vacuum conditions ($p<10^{-5}$ mbar). A part of the 10 h heated substrates was reheated for 48 h (will be denoted as 10 + 48 h). Ru-Al thin films were deposited on pieces of each kind of preheated substrate by magnetron co-sputtering from elemental Ru and Al targets (for details of deposition see [7]).

The substrate-film systems have then been again annealed under high vacuum conditions at 800 ${}^{\circ }$C for 10 h. The phase formation of the annealed RuAl films has been investigated by X-ray diffraction (XRD, Philips X’Pert PW3040/00, Co-K*α*, PANalytical, Almelo, The Netherlands) in Bragg Brentano geometry.

The surface of the substrates and substrate-film systems was analyzed by light microscopy (LM, Zeiss Axiotech HAL100, Carl Zeiss Microscopy GmbH, Jena, Germany), scanning electron microscopy (SEM, Zeiss Ultra Plus, Carl Zeiss Microscopy GmbH, Jena, Germany) and atomic force microscopy (AFM, DI Dimension 3100, Bruker (formerly Digital Instruments), Billerica, MS, USA). Cross sections have been prepared by the focussed ion beam technique (FIB, Zeiss 1540 XB CrossBeam, Carl Zeiss Microscopy GmbH, Jena, Germany).

High-angle annular dark field scanning transmission electron microscopy (HAADF-STEM, FEI Technai F30, FEI, Hillsboro, OR, USA) was performed to analyze the microstructure on the nanoscale. Together with energy dispersive X-ray spectroscopy (EDX, EDAX, Mahwah, NJ, USA) in the same instrument the local chemical composition has been determined.

## 3. Results and Discussion

### 3.1. Substrate Morphology

**Figure 1:**
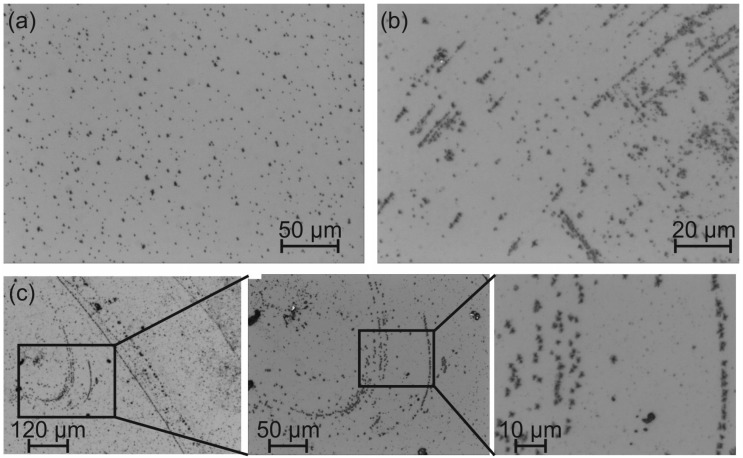
Light microscope image of the 48 h preheated La${}_{3}$Ga${}_{5}$SiO${}_{14}$ (LGS) substrate showing (**a**) a region with homogeneous distribution; and (**b**) the agglomeration of the features along scratches at the sample surface; (**c**) Overview image the 10 + 48 h LGS substrate together with zoom images showing agglomeration of the features at scratches.

The light microscope images (Figure 1) show an overview of the substrate surface of the LGS heated for 48 h. A lot of small features are visible. Figure 1a demonstrates a region with an isotropic distribution of the features while Figure 1b shows an accumulation at scratches at the sample surface. The overview image of the 10 + 48 h LGS substrate in Figure 1c shows neighboring sample regions with homogeneous distribution of the features and with agglomeration at the scratches. The zoomed images clarify that the occurrence of the features follows the path of the scratches. This observation leads to the assumption that the features preferably form at points where there is a damage or inhomogeneity in the single crystal lattice.

**Figure 2:**
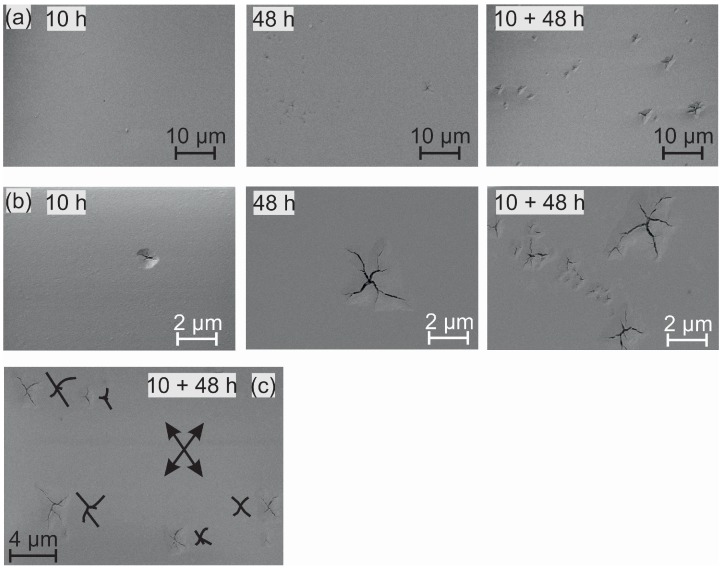
SE-SEM images of the heated substrates at low (**a**) and high (**b**) magnification. (**c**) SE-SEM image of the 10 + 48 h sample. The arrows indicate the two main directions of the cracks in the substrate.

To reveal the nature of the features, investigations with scanning electron microscopy have been performed. Images with two different magnifications are presented in Figure 2a,b. The high magnification image clarifies that the features are cracks in the surface of the substrate. For the short heating time of 10 h, hardly any changes are found on the surface, only very few small cracks appear. For the longer heated samples, the area density of such cracks strongly increases. As it can be seen in the higher magnification images in Figure 2b, also the size of these features becomes larger with increasing heating time. Additionally it appears that the alignment of the cracks at the substrate surface is not random. In most of the features, the direction of the cracks in the surface is quite the same, as is indicated by the arrows in Figure 2c. In this image, for a better visibility the direction of the cracks is outlined by the sketch next to the features. It has to be assumed that there is a correlation with the crystal structure.

LGS crystals were developed about 30 years ago. It is known that these crystals are not completely homogeneous, so differences in composition and properties occur [11]. Ga and Si may occupy same positions and also oxygen vacancies are present. These variations in composition also lead to differences in the color of the LGS crystals. Kuzmicheva, *et al.* showed that in Czochralski-grown LGS crystals the composition may vary both axially and radially [12]. They also showed the coexistence of two isostrucutral solid solutions [12]. Capelle, *et al.* summarize that the dominant inhomogeneities in LGS are dislocations, growth bands and inclusions and that recently crystals with a reduced density of dislocations and inclusions were prepared [11]. Wang and Uda investigated the homogeneity of LGS with a bulk-wave-measurement and showed that the sound velocity varies across the wafer. They also conclude that there is a need for an improvement of the crystal growth [13]. In summary, there exist a lot of inhomogeneities which can support the formation of diffusion channels and the development of such cracks. Moreover, inhomogeneities which are introduced from outside, like small scratches from the sawing of the wafers might additionally promote the formation of the observed features.

**Figure 3:**
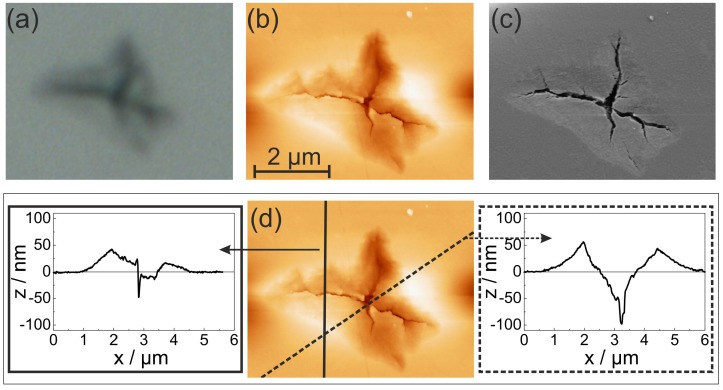
(**a**) Light microscope; (**b**) atomic force microscopy (AFM); and (**c**) SE-SEM image of the same crack of the 10 + 48 h pretreated LGS substrate; (**d**) shows the AFM line profiles across the crack.

A comparison of light microscopy, atomic force microscopy and scanning electron microscopy of the same sample position (sample: 10 + 48 h) is presented in Figure 3. The AFM image reveals the complicated surface morphology with elevations and subsidences of the surface around the crack. This can be quantified by line profiles of the AFM image, as presented in Figure 3d. At the outer region of the structure, the surface is raised up to 60 nm, followed by a slope to the center of the crack. The deepest point measured by AFM of about minus 100 nm is of course limited by the geometry of the AFM tip and doesn’t reproduce the actual depth.

**Figure 4:**
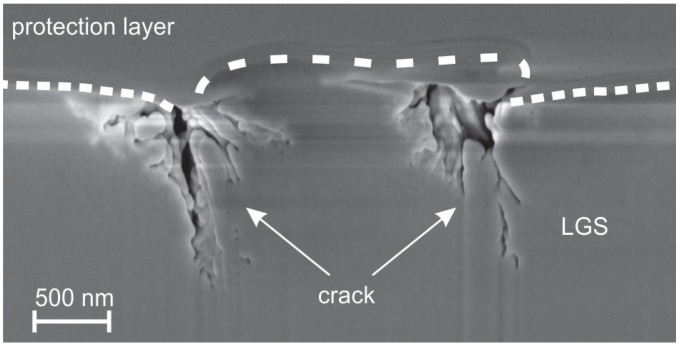
In-lens SEM image of a crack in the 10 + 48 h heated LGS substrate. The dashed line serves as guide to the eye for the interface between LGS substrate and protection layer which was deposited for the FIB process.

To get more information about the real depth of the cracks, cross sections were analyzed. An SEM in-lens image of such a cross section of a crack is presented in Figure 4. It shows that these cracks are extended to a huge network of channels below the surface of the substrate. They reach a depth of more than 1 μm. This finding resembles the formation of voids near the LGS substrate surface of a SAW device in uncovered areas between the finger electrodes after a heat treatment at 650 ${}^{\circ }$C in light vacuum (0.4 mbar) observed by Bardong, *et al.* [10].

### 3.2. Influence of LGS Annealing on RuAl Thin Films

As an example, the LGS 10 + 48 h pretreated substrates covered with a 110 nm RuAl metallization will be discussed in this section.

**Figure 5:**
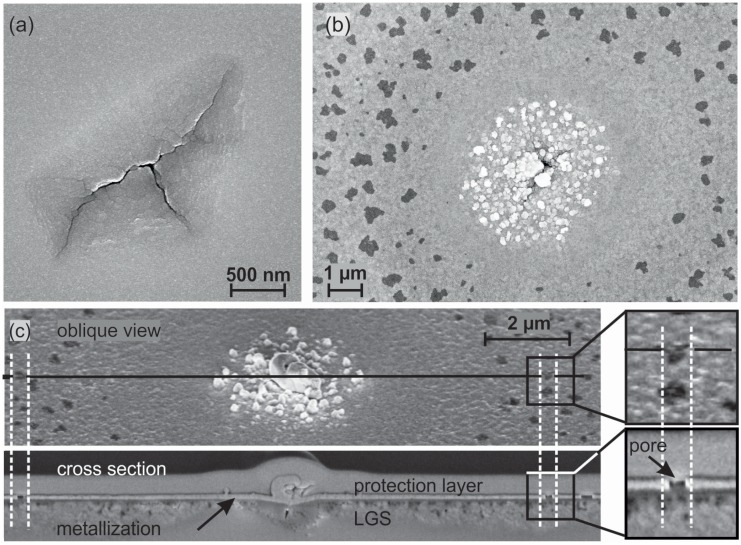
In-lens SEM image of a RuAl thin film deposited on a pretreated LGS substrate (**a**) before; and (**b**) after annealing at 800 ${}^{\circ }$C under high vacuum conditions at the position of a crack in the LGS (both images not showing the same sample position); (**c**) In-lens SEM image of such a feature taken in the FIB device together with an In-lens SEM image of a FIB cut through it.

Figure 5a shows an image of such a sample in the as-deposited state of the metallization. A homogeneous smooth film with fine grains has formed and the crack is clearly visible. The morphology of the film strongly changes after annealing at 800 ${}^{\circ }$C for 10 h under high vacuum (Figure 5b, different sample position).

Clearly visible is the crack in the center of the image, but the metallization is not homogeneous any more. The crack is surrounded by bright structures which seem to be grown out of the film. Around this area, there is a homogeneous ring followed by an area with dark structures. Figure 5c shows an image of such a feature taken in the FIB device together with a FIB cross section across the center of the crack, the homogeneous ring around it and through the region with the dark spots. In the center, huge structures have grown out of the film. Around the center, the metallization is continuous. In larger distance to the center, it can be seen that at the positions where the FIB cut goes through such a dark structure there is a pore within the metallization. Former investigation of RuAl films directly deposited on untreated LGS substrates and annealed at 800 ${}^{\circ }$C proved a complete demixing of the RuAl alloy in an Al${}_{2}$O${}_{3}$ layer above the substrate, followed by a nearly pure Ru layer and with a thinner Al${}_{2}$O${}_{3}$ layer on top [14]. Pores were found within the Ru layer, while both Al${}_{2}$O${}_{3}$ layers are continuous. The same behaviour is now determined for the film in a distance to the cracks, while near to the cracks there are no pores.

**Figure 6:**
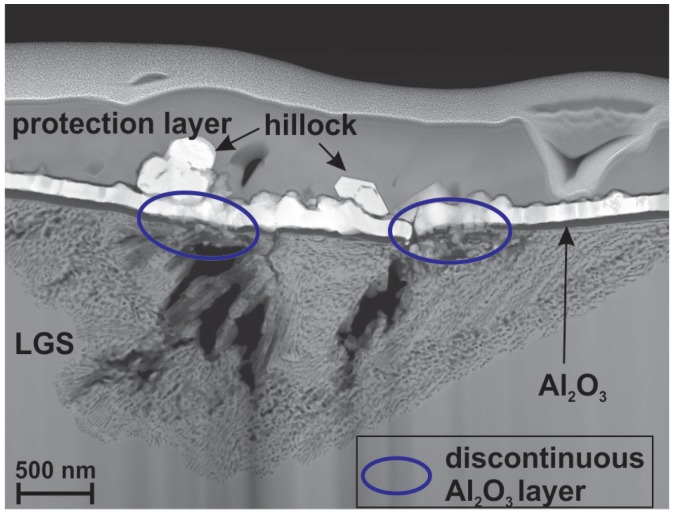
High-angle annular dark field scanning transmission electron microscopy (HAADF-STEM) image of the RuAl film on the pretreated LGS substrate (10 + 48 h) after annealing at 800 ${}^{\circ }$C for 10 h in high vacuum.

Figure 6 shows the high-angle annular dark field scanning transmission electron microscopy image of a cross section of such a structure. The large cracks are clearly visible. They are surrounded by a huge network of finer channels. At this position, the network has a depth of 1.6 μm and a width of about 4 μm. Above this channel-region, huge hillocks grow out of the metallic film. Although the metallization was deposited with a thickness of about 110 nm, these hillocks are up to 400 nm high.

**Figure 7:**
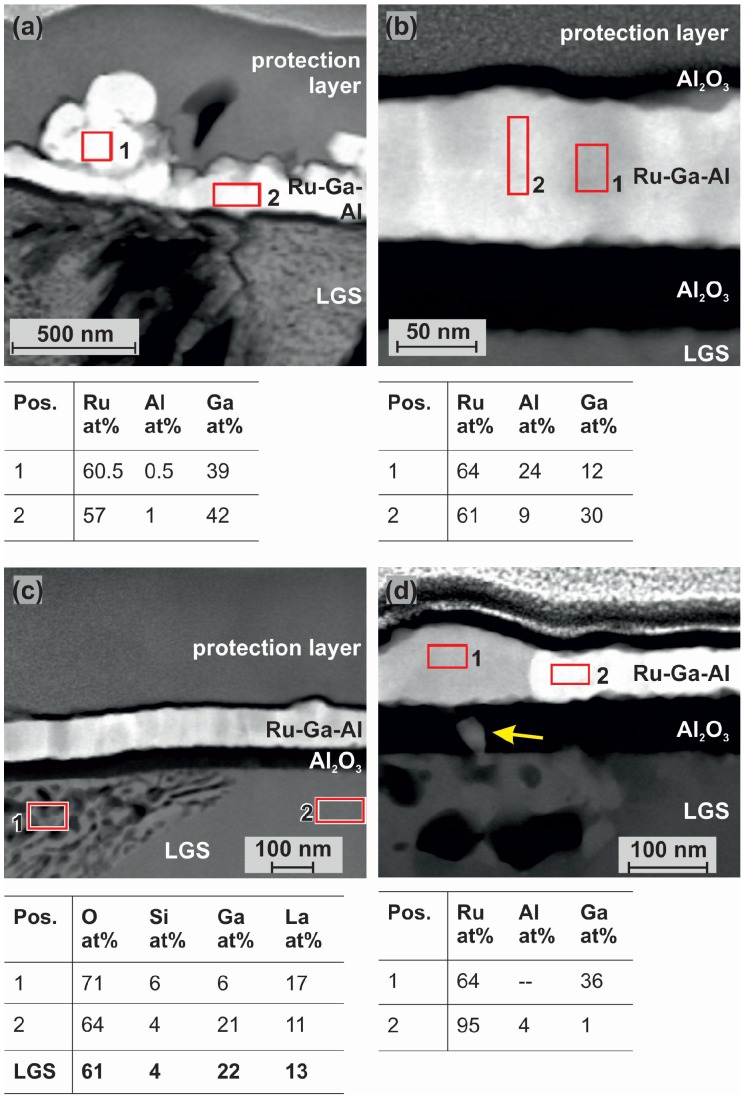
Energy dispersive X-ray spectroscopy (EDX) measurements of the RuAl film deposited on the pretreated substrate after annealing at 800 ${}^{\circ }$C for 10 h. (**a**) at a position at the center of the crack; and (**b**) near to the crack; (**c**) EDX measurement of the LGS substrate in the region of the channels and in an undisturbed region; (**d**) EDX measurement of the RuAl film at a position where the Al${}_{2}$O${}_{3}$ layer is disturbed. The yellow arrow indicates an inhomogeneity within the Al${}_{2}$O${}_{3}$ layer.

It can be seen that an Al${}_{2}$O${}_{3}$ layer has formed between the substrate and the metallic film, which is continuous in larger distance to the cracks (see right region of the image), but which is disturbed and not continuous directly at the position of the cracks. These positions are marked with the blue circles. It is obvious, that the hillocks form exactly at those positions where the Al${}_{2}$O${}_{3}$ layer is discontinuous. The EDX measurements show that the hillocks have a composition of about 60 at% Ru and 40 at% Ga (Figure 7a). Above the zone of the channel network, the metallic layer also has the composition of 60 at% Ru, while the residual 40 at% are partly Ga and Al (Figure 7b). As was shown by Seifert, *et al.* for RuAl films deposited on thermally oxidized Si besides Ru rich grains, a Ru${}_{60}$Al${}_{40}$ phase was most dominant after annealing at 800 ${}^{\circ }$C under high vacuum conditions [14]. For the films now prepared on the pretreated substrates, we find the composition Ru${}_{60}$Al${}_{x}$Ga${}_{40-x}$. Although no phase diagram is known for the system Ru-Ga, several Ru-Ga phases have been determined. Among them exists the RuGa phase, which has the same crystal structure like RuAl [15]. Altogether, this leads to the assumption that if the Al${}_{2}$O${}_{3}$ layer on top of the LGS substrate is not continuous, it allows a strong diffusion of Ga from the substrate into the upper metallic film. There, Ga replaces Al within the RuAl phase, which is oxidized by O diffusing out of the substrate and forming the Al${}_{2}$O${}_{3}$ layer at the interface to the substrate.

In a larger distance to the cracks, a complete oxidation of the Al atoms and the formation of a nearly pure Ru layer is similarly found for the samples prepared on the pretreated substrates as compared to the untreated ones [14]. As an example, Figure 7d illustrates that only where the Al${}_{2}$O${}_{3}$ layer is disturbed (see the bright contrast in Al${}_{2}$O${}_{3}$ layer on the left hand side marked by the yellow arrow), a large amount of Ga diffuses into the Ru film and forms the RuGa phase. Close beside this point, the Al${}_{2}$O${}_{3}$ layer is intact (right hand side), hardly allowing Ga to diffuse into the Ru film, resulting in a chemical composition of the metallic layer of 95 at% Ru. The presence of small amounts of Ga in the Ru layer which formed after annealing a RuAl alloy on an untreated LGS substrate (under high vacuum conditions for 10 h at 800 ${}^{\circ }$C) was already shown in [7].

The above described finding that there are no pores in the film near to the cracks but that pores are formed in larger distance to the cracks could be explained in the following way: the diffusion of Ga out of the substrate in the region of the cracks leads to the formation of a Ru-Al-Ga phase, whereas in larger distance to the cracks there is hardly any diffusion of Ga. In former work, the formation of pores was described as a stress relaxation mechanism due to the outwards diffusion of the Al atoms [14]. Due to the inwards diffusion of Ga these stresses do not occur and no pores are formed—in contrast to the positions where the RuAl layer has demixed and no Ga has diffused into the film. Therefore, the pores are only found in larger distances to the cracks.

The analysis of the substrate composition in the range of the channels and in an undisturbed area shows that the Ga content is strongly decreased in the first case (Figure 7c). This is consistent with the large Ga content within the metallic film. At farther distance from the cracks, the composition of the LGS corresponds quite well to the stoichiometric value.

**Figure 8:**
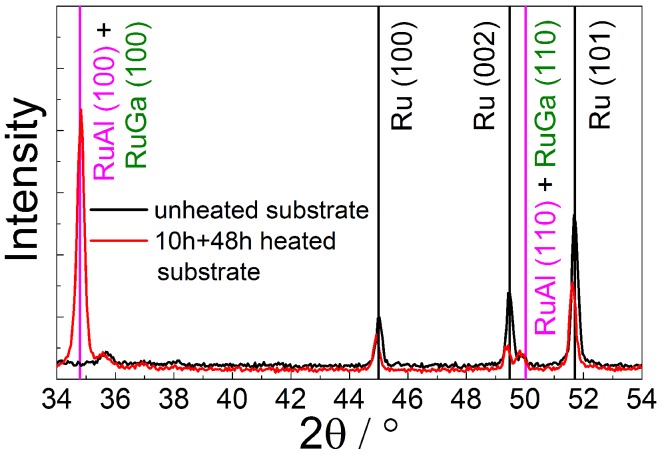
X-ray diffraction (XRD) measurement of a RuAl thin film deposited on an unheated (black) and on a 10 + 48 h heated (red) LGS substrate after 10 h annealing at 800 ${}^{\circ }$C at high vacuum.

The XRD measurement of the heated RuAl sample on the 10 + 48 h pretreated substrate (Figure 8) shows a peak at the position of the RuAl (100) reflex in contrast to a RuAl sample deposited on an untreated substrate and annealed at 800 ${}^{\circ }$C. In the latter case, the RuAl (100) is not present since all Al is oxidized and only a pure Ru layer has formed [7]. Therefore, only Ru-reflexes appear in the XRD measurement. In contrast to this, as described above (Section 3.2), the film on the pretreated substrate shows a chemical composition of Ru${}_{60}$Al${}_{x}$Ga${}_{40-x}$ in the regions with the disturbed substrate and there is no complete demixing in Al${}_{2}$O${}_{3}$ and pure Ru. Since RuAl and RuGa possess the same crystal structure, the strong reflex at 34.8${}^{\circ }$ as well as the smaller reflex at 49.9${}^{\circ }$ might be caused by a RuAl, RuGa or Ru-Al-Ga phase.

## 4. Conclusions

We conclude that a preheating of LGS substrates under vacuum conditions leads to damages within the substrate. A network of channels develops, which simplifies the outwards diffusion of Ga out of the substrate. Significant amounts of Ga are found within the deposited Ru-Al layer after a further heat treatment. A pretreatment is not suitable to reach an equilibrium state in the LGS to reduce an oxidation of the deposited film. Additionally, the results show the huge challenges which will occur if LGS is applied at high temperatures under vacuum conditions or reducing atmospheres. Such an application will not be possible without additional protection layers.
